# Mortality during the first four waves of COVID-19 pandemic in Israel: March 2020–October 2021

**DOI:** 10.1186/s13584-022-00533-w

**Published:** 2022-05-31

**Authors:** Ziona Haklai, Nehama Frimit Goldberger, Ethel-Sherry Gordon

**Affiliations:** Division of Health Information, Ministry of Health, Jerusalem, Israel

**Keywords:** Excess mortality, COVID-19, COVID vaccinations, Non-COVID-19 mortality

## Abstract

**Background:**

The COVID-19 pandemic caused four waves of infection in Israel until October 2021. Israel was the first country to offer vaccinations to all the adult population followed by boosters. This study addresses how mortality rates reflect the effects of the pandemic.

**Methods:**

Total mortality rates and rates of mortality without COVID-19 deaths (non-COVID-19 mortality) between March 2020 and October 2021 were compared with the average pre-COVID-19 rates in 2017–2019 by month, population group and by age group. In addition, a cohort vaccinated at least once by 31 March 2021 was followed up for mortality in the following seven months compared to the corresponding months in 2017–2019.

**Results:**

A small number of excess deaths was found in the first wave and a greater excess in the following waves. The monthly mortality rate ratio was highest in October 2020, 23% higher than the average in 2017–2019, followed by August 2021 (22%), September 2021 (20%) and September 2020 (19%). Excess mortality in the Arab population was greater than for Jews and Others, and they had 65% and 43% higher mortality in September and October, 2020, 20–44% higher mortality between December 2020 and April 2021 and 33%, 45% and 22% higher mortality in August, September and October 2021, respectively. In most months of the pandemic, the non-COVID-19 mortality rates were not significantly different from those in 2017–2019. However, between November 2020 and March 2021, they were significantly lower for the total population and Jews and Others. They were significantly higher for the total population only in August 2021, and particularly for the Arab population. Non-COVID-19 mortality was also lower for most sex/age groups over the total study period. In a cohort of 5.07 million Israeli citizens vaccinated at least once by 31 March, 2021, age adjusted and age specific mortality rates for the following 7 months were lower than the average rates in 2017–2019 for these months,

**Conclusion:**

Israel has seen significant excess mortality during the COVID-19 pandemic, particularly in the Arab sector. Following lockdowns and administration of vaccinations excess mortality was reduced, and no excess mortality was seen amongst the vaccinated in the months after the vaccination campaign. These findings highlight the importance of public health measures such as mandating mask wearing and population vaccinations to control infection and reduce mortality.

**Supplementary Information:**

The online version contains supplementary material available at 10.1186/s13584-022-00533-w.

## Background

The pandemic due to the novel severe acute respiratory syndrome coronavirus2 (SARS-CoV-2, COVID-19), has brought five major waves of infection to Israel, each resulting in hospitalizations of severe cases and deaths of confirmed cases. The first wave began when the emerging virus reached Israel in March 2020 and lasted until May 2020, the second wave was between June and October 2020 and the third wave started in November 2020. In a previous paper on excess mortality in Israel between March and October, 2020, [1], the excess mortality was presented by month, age, population group and locality for the first two waves. A recent paper by Peretz et al. [2] analyzed total excess mortality in Israel by age groups until the end of March 2021, using a predictive model based on 19 years of mortality data (2000–2019) and including weather data, influenza counts, and yearly population sizes, and comparing excess mortality with that attributed to COVID-19. They found 12% higher excess for the whole period analyzed, with varying excesses in different age groups over age 19.

Many significant changes have occurred during 2021. Israel was the first country to provide vaccinations for all the adult population. The Pfizer BNT162B2 vaccine was given, starting on December 19, 2020, first to medical personnel and others at high risk, followed by the older population aged 60 and above, and then continuing with the rest of the adult population by decreasing age groups. This lead to a rapid decrease in the incidence of coronavirus disease (COVID-19) from February 2021. The short term efficacy of the vaccine in Israel in reducing mortality has been reported [3], but not a long term mortality follow up of vaccinated persons.

The emergence of the delta variant in India in early 2021 which became the dominant variant worldwide, together with the waning protection of the vaccine, led to a fourth wave which started in Israel in June 2021. In response to this, a first booster shot was administered to the public starting at the end of July 2021, which helped to bring this wave under control by November 2021. Starting in December 2021, the omicron variant together with waning protection of the booster has led to a fifth wave.

Excess mortality is an important indicator of the mortality effects of the pandemic as it reflects both deaths occurring as a direct result of the virus and the indirect effects on mortality of the pandemic such as from lockdown measures, interruption of standard medical care and pressure on the health system due to COVID-19 hospitalization or late effects of COVID-19 infection. Total mortality is also independent of measurement of COVID-19 infection, which may be under-estimated by lack of testing due to low availability of tests or non-compliance of the population with the requirement to test.

Israel’s Ministry of Health maintains a database of persons who tested positive for the COVID-19 virus, and collates data from various sources including hospitals, long-term care facilities, geriatric homes and other community sources of persons who died due to COVID-19. These were classified as COVID-19 deaths for our analysis, although this definition is provisional as final coded cause of death data based on the death notification forms is not yet available. All other deaths were classified as non-COVID-19.

In this study, we followed the total mortality rates and non-COVID-19 mortality rates in Israel between March 2020 and October 2021 compared to the average rates in 2017–2019, to assess trends in excess mortality, and COVID-19 and non-COVID-19 mortality by month and population group, and by age and population groups for the total period. In addition, we followed-up a cohort of vaccinated persons to compare their mortality to corresponding months during 2017–2019.

## Methods

Total mortality data in Israel was obtained from the Ministry of the Interior to which all deaths in Israel are reported by law. Population estimates were received from the Central Bureau of Statistics. We compared crude death rates between March 2020 and October 2021 with the average during 2017–2019, by month, population group and age group. The population in Israel is generally grouped into Jews and Others (non-Jewish residents who are not Arabs) and Arabs (including Moslems, Christian Arabs and Druze), the groups we used here. In this paper, we report the ratio of both total mortality rates and of non-COVID-19 mortality rates compared to rates in 2017–2019. Each population group, age group, sex and month during 2020–2021 was compared with corresponding ones in 2017–2019. We assumed that the age composition of each population group and of the total population remained constant during 2017–2019 and the pandemic period and therefore age-adjustment was not needed for the all-age rate ratios.

We merged the Ministry of Health database of COVID-19 vaccinations and verified COVID-19 cases with the Ministry of Interior death data to follow a cohort of persons aged 15 and over who had at least one vaccination by March 31, 2021, for mortality during the next 7 months (April–October, 2021), and compared their age- specific and age-adjusted mortality rates with that of the total population in these months in 2017–2019. The standard population used was the total population of Israel in 2019, with age groups of 15–24, 25–44, 45–54, 55–64, 65–74, 75–84 and 85 and above.

We used a Fisher exact test to determine the significance of the rate ratios. Results with a *p*-value was ≤ 0.05 were considered significant. The analysis was done using SAS 9.4.

We also present infection rates by month to compare with mortality, calculated from the Ministry of Health COVID-19 database, age-adjusted to the 2009 Israeli population and for age 65 and above, for the total population, and for total Jewish and Arab localities. Only place of residence was available from this database, so it was used as a proxy for population group since most of the two groups live in separate cities.

## Results

Between March 2020 and October 2021 there were 84,124 deaths in Israel giving an excess of 8953 deaths compared to the average of 2017–2019, slightly higher than the number of deaths attributed to COVID-19 in this period, 8114.

### Mortality by month and population group

**Fig. 1:**
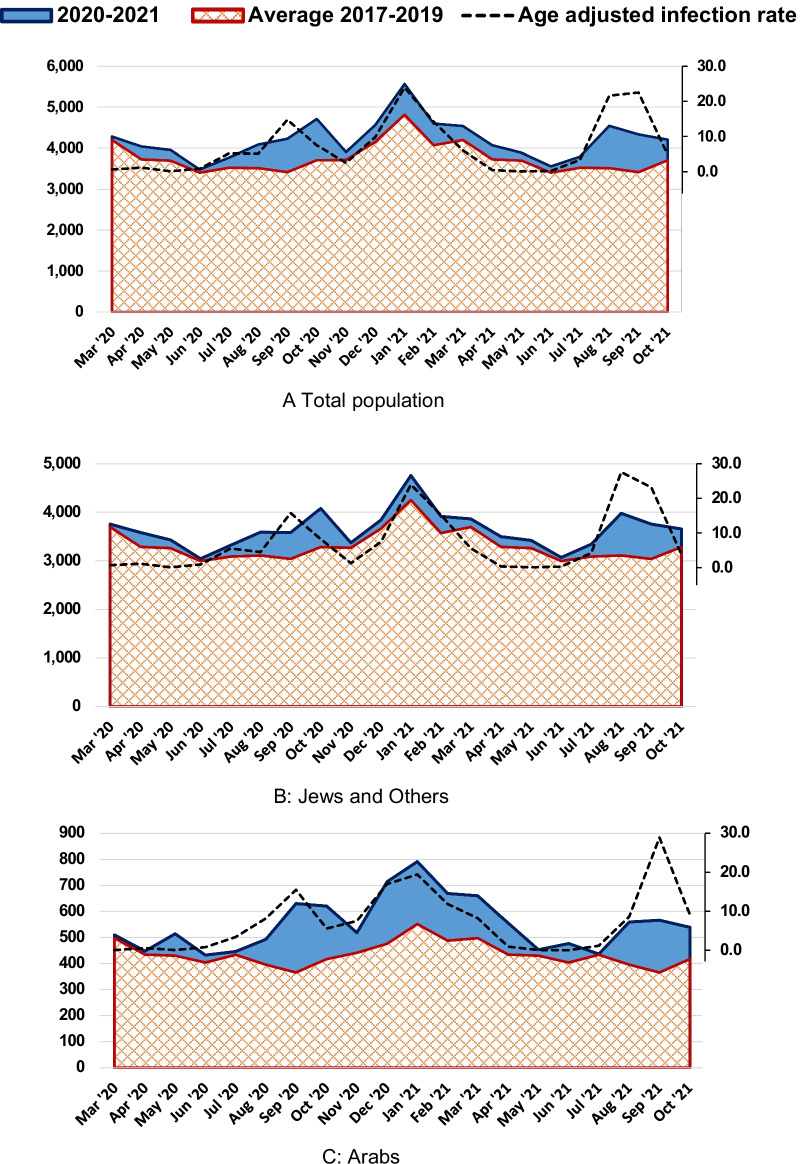
Number of deaths in COVID-19 pandemic period compared to average in 2017–2019. Total number of deaths in March 2020-October 2021 compared to average in 2017–2019, by month and population group. Age-adjusted infection rate per 1000 persons in all localities (**A**), and Jewish (**B**) and Arab (**C**) localities is shown as a dashed line, according to the secondary scale on the right hand side. Adjusted to the 2009 Israeli population as standard population

Figure 1 shows the monthly number of deaths in Israel between March 2020 and October 2021 compared to the average for these months in 2017–2019 for the total population, Jews and Others, and Arabs. The dashed line shows the age-adjusted infection rate per 1000 persons, according to a secondary scale on the right. Table 1 shows the ratio of corresponding mortality rates, and Additional file 1: Table S1 gives their confidence intervals (CI). We see a small number of excess deaths in the first wave between March and June, 2020, and a much greater excess with highly significant rate ratios in the following waves between August and October, 2020, December 2020-February 2021 and August-October, 2021. The mortality profile is similar to the infection rate, although in the second wave there was a lag between the peak of infection in September 2020 and mortality, and in the fourth wave, the peak of infection in September 2021 was higher than the mortality peak, unlike earlier waves.

**Table 1:**
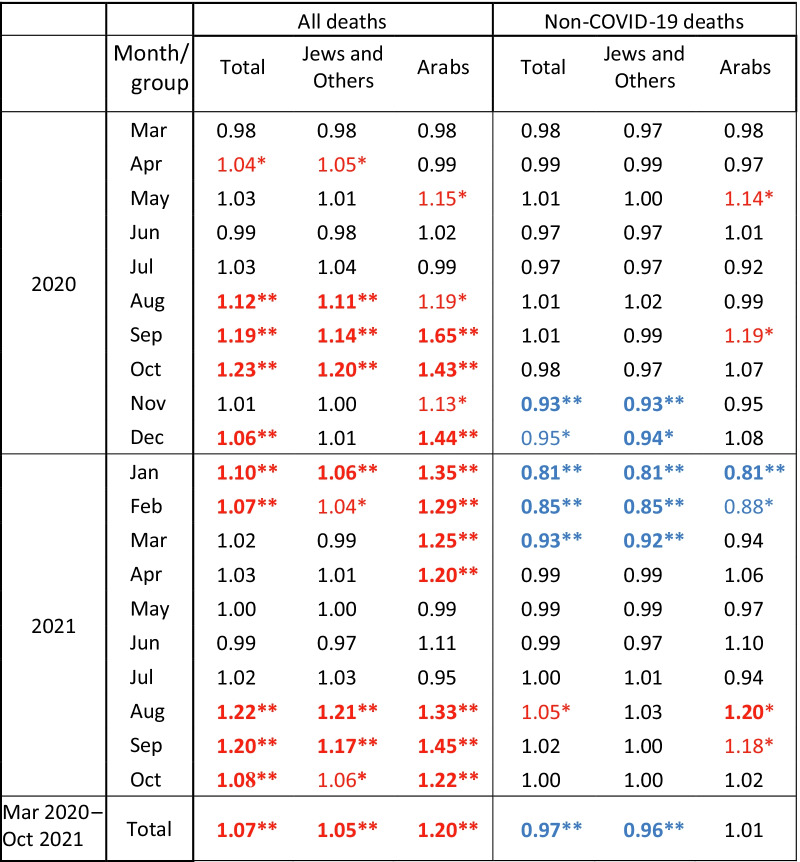
Ratio of mortality rates for the period March 2020–October 2021, for all deaths and non-COVID-19 deaths, compared to average of 2017–2019, by month and population group

Significance of rate difference: **p ≤ 0.001, bold (red = high, blue = low), *0.001 < *p* ≤ 0.05 not bold (red = high, blue = low)

The monthly mortality rate ratio was highest in October 2020 with a mortality rate 23% (95% CI 18–27%) higher than the average in 2017–2019, but it was almost as high in August 2021 (22%, [18–27%] higher), September 2021 (20% [16–24%] higher) and September 2020 (19% [15–24%] higher). Excess mortality in the Arab population was greater than for Jews and Others. Highest excess rates were in the second wave for the Arab population, reaching 65% (95% CI 50–82%) higher mortality in September 2020 and 43% (95% CI 30–57%) in October 2020. The third wave of COVID-19 was very long in the Arab population and they had significantly higher mortality rates for 5 months, 44% (32–57%) higher in December 2020, and 35% (24–46%) in January 2021 gradually decreasing to 20% (9–33%) in April 2021. In the fourth wave, the Arab excess mortality was higher than for Jews and Others, with mortality rates 33% (20–47%), 45% (31–61%) and 22% (8–33%) higher in August, September and October 2021, compared to 21% (17–26%), 17%, (13–22%) and 6% (1–9%), respectively, for Jews and Others.

**Fig. 2:**
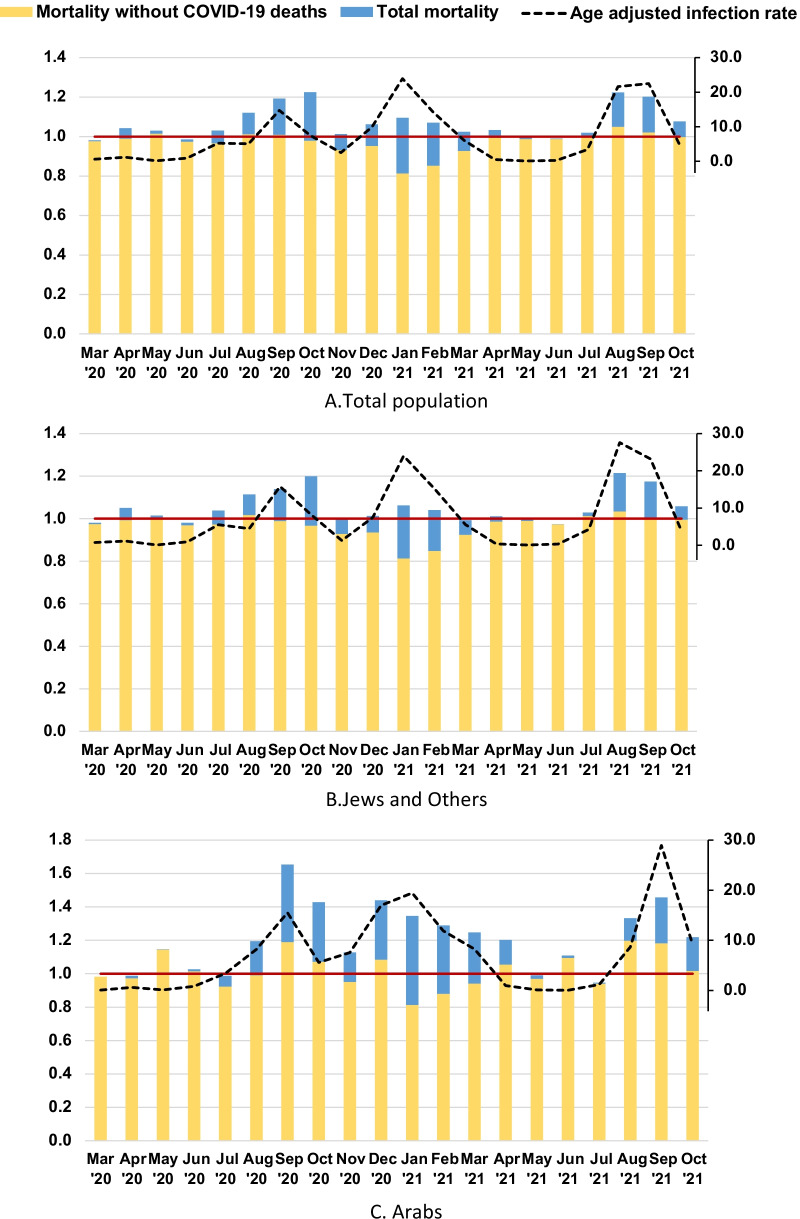
Ratio of mortality rates, COVID-19 pandemic period compared to 2017–2019, all deaths and non-COVID-19 deaths. Ratio of mortality rates in March 2020-September 2021 compared to average in 2017–2019, for all deaths and non-COVID-19 deaths, by month. Age-adjusted infection rate per 1000 persons in all localities (**A**), and Jewish (**B**) and Arab (**C**) localities is shown as a dashed line, according to the secondary scale on the right hand side. Adjusted to the 2009 Israeli population as standard population

Figure 2 shows the ratios of mortality without COVID-19 deaths (non-COVID-19 mortality) compared to 2017–2019, superimposed on the corresponding ratio for total mortality, for total population, Jews and Others and Arabs. These rate ratios are presented in Table 1, and CI in Additional file 1: Table S1. The infection rate is shown as in Fig. 1 by the dashed line.

In most months of the pandemic, the non-COVID-19 mortality rates were not significantly different from those in 2017–2019. However, between November 2020 and March 2021, they were significantly lower for total population and Jews and Others, and hence the total excess mortality was less than that expected from the high COVID-19 mortality. Only in August 2021 was the rate of non-COVID-19 mortality significantly higher than 2017–2019, 5% (95% CI 1–9%) higher for the total population and a very significant 20% (8–33%) higher for the Arab population. In the Arab population the non-COVID-19 mortality was also significantly higher in May and September 2020 and September 2021, by 14% (3–27%), 19% (7–33%) and 18% (6–32%), respectively.

### Mortality by sex and age group

Table 2 shows the ratio of mortality rates for the period March 2020–October 2021, for all deaths and non-COVID-19 deaths, compared to the average rates for parallel months in 2017–2019, by age groups, sex and population group. 95% confidence intervals for the table are in Additional file 1: Table S2.

**Table 2:**
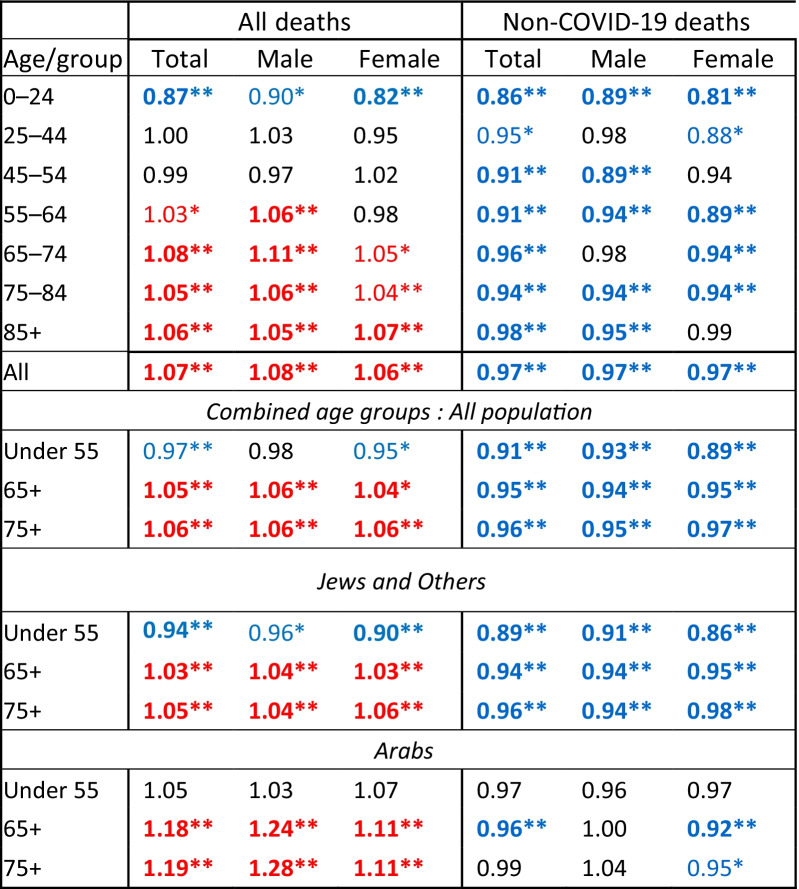
Ratio of mortality rates for the period March 2020–October 2021, for all deaths and non-COVID-19 deaths, compared to average of 2017–2019, by age groups, sex, and population group

Age 65 and above in the combined age groups includes 75 and above

Significance of rate difference: **p ≤ 0.001, bold (red = high, blue = low), *0.001 < *p* ≤ 0.05 not bold (red = high, blue = low)

Total mortality rates were significantly higher by 7% (95% CI 6–8%) during the pandemic period, by 8% (7–9%) for males and 6% (5–7%) for females, than the average of 2017–2019, and significantly higher rates were similarly found for age groups over 65, over 75 and over 85 (6% [5–7%], 6%[4–7%] and 5% [3–7%] in males and 4% [2–5%], 6% [5–8%] and 7% [6–9%] in females, respectively). An even higher excess was found for males aged 65–74, 11% [8–13%] compared to 5% [2–8%] for females. No significant excess mortality was found for younger age groups, 25–44 and 45–54 and the combined under 55 group, and in fact, mortality rates were significantly lower by 13% (8–17%) at ages 0–24. Non-COVID-19 mortality was significantly lower for the period of March 2020–October 2021 than the average of in 2017–2019 in most age groups, and lower in females than males in groups under age 44 and aged 55–64.

The Arab population had higher excess mortality than Jews and Others, for groups aged over 65 and aged over 75, 24% (20–29%) and 28% (23–34%), respectively in males and 11% (7–15%, 6–16%) for both groups in females, compared to between 4% (2–5%) and 6% (4–7%) for Jews and Others.

### Mortality of a vaccinated cohort

We followed 5.07 million Israeli citizens who had been vaccinated at least once by March 31, 2021. 19,457 of them died in the following 7 months, April–October 2021, 4.4% (855) of which were recorded as COVID-19 deaths. Of the remaining deaths in Israel in this period, 11.3% were COVID-19 deaths. The ratio of the vaccinated cohort’s mortality rates compared to the average mortality rates for these months in 2017–2019 is shown in Table 3 (95% CI in Additional file 1: Table S3), by age and sex and for total age adjusted rates. Mortality rates for those vaccinated in 2021 were lower than the 2017–2019 average, significant at most ages. Total age adjusted mortality in this cohort was 14% (95% CI 12–15%) lower, 13% (11–15%) in males and 16% (14–18%) in females. Age specific mortality ratios were lowest for ages 45–54 in males (0.76, 95% CI 0.69–0.85) and 55–64 (0.70, 0.64–0.77) in females. The mortality ratios for these seven months in the vaccinated were also lower than those for the whole pandemic period presented in Table 2 at all ages, and for ages 55 and over showed lower rather than excess mortality.

**Table 3:**
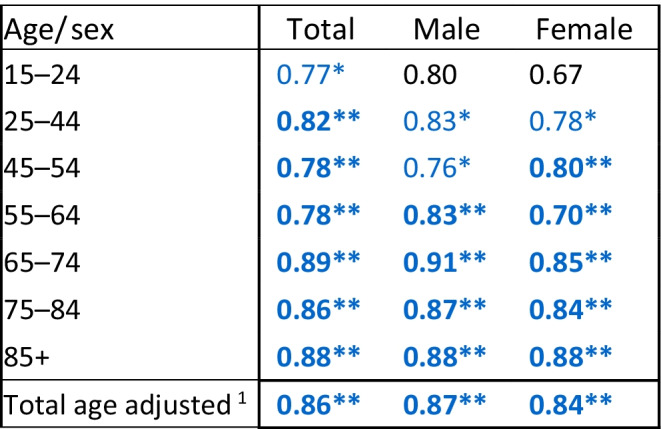
Ratio of mortality rates for the period April–October 2021 of a cohort of persons vaccinated before 31 March 2021, aged 15 and above, compared to the average mortality rates in April–October 2017–2019, by age and sex

Significance of rate difference: **p ≤ 0.001, bold (blue = low), *0.001 < *p* ≤ 0.05 not bold (blue = low)

^1^Adjusted to population of Israel in 2019 as standard population

### Infection rates in Jews and Others compared to Arabs

**Fig. 3:**
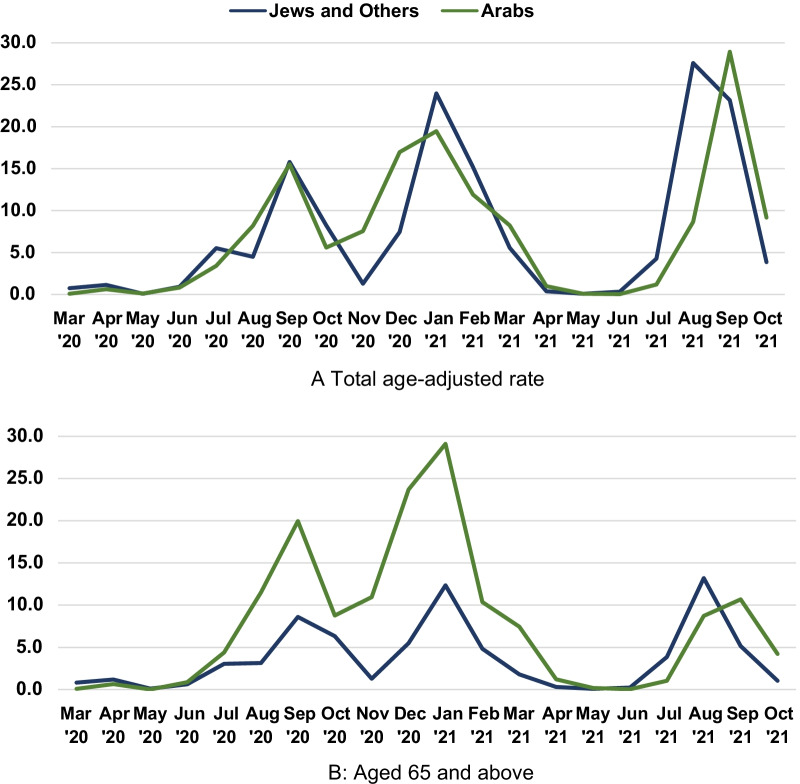
Rate of COVID-19 infection per 1000 persons by month by population group. Rate of COVID-19 infection per 1000 persons, age-adjusted to the 2009 Israeli population as standard population (**A**), and at age 65 and above (**B**), in all Jewish compared to all Arab localities in Israel by month

Although age-adjusted infection rates of Jews and Others and Arabs were similar (Fig. 3A), with slightly lower rates peak rates for Arabs in the third wave (December 2020–March 2021), rates at age 65 and above were much higher in the second and third waves peaking at rates over twice as high.

## Discussion

In this study of excess mortality during the COVID-19 pandemic in Israel, we show significantly high excess mortality in the months of peak infection. Our method of comparison of mortality with previous 3-years’ average allowed analysis by population group, age groups, for a vaccinated cohort, and similar comparison of non-COVID-19 mortality. By calculating rate ratios and comparing corresponding months, we controlled for changes in population size and for seasonality, although not for decreasing mortality trend and influenza morbidity, included in the more refined statistical model of Peretz et al. [2]. However, they noted a smaller decrease in mortality in recent years, so this effect is likely to be small.

We found higher excess mortality among Arabs then Jews and Others, and significant only in the older population aged 55 and above.

### Public health measures and their effect

During the first two waves of the pandemic, the Israeli government mandated strict containment measures starting with limiting public gatherings and celebrations, limited use of public transport, closing of non-essential shops, closing of schools and places of worship and finally lockdowns. Mask wearing was required from 12 April. Lockdowns were in place between 8 April and 3 May, and between 18 September and 17 October, with some restrictions remaining afterwards and in selected areas.

We cannot prove a direct connection between these measures and mortality. It would also be expected to have a lag between the decrease in infections and decrease in excess mortality, as in general infected patients were first hospitalized and deteriorated for a few weeks until they unfortunately succumbed to the virus or its complications. But we can see the swift decrease both in infections and excess mortality following strict lockdowns.

In the first wave, the excess mortality in Israel was low compared to many other countries [1, 4], and by May 2020 there was no significant excess in the total population and Jews and Others. This may be attributed to the strict and speedy restrictions and lockdown. The second wave which led to high excess mortality in August-October 2020, including the highest monthly excess mortality found in the pandemic, appears to have been halted by the second strict lockdown with no significant excess in the following month, November, 2020.

The third wave led to high excess mortality in December 2020–February 2021, particularly high among Arabs until April2021. There were lockdown measures in January–February 2021, less strict than in earlier waves, but in addition there was a swift and comprehensive vaccine program. These together appear to have led to a speedy reduction in infections and subsequent mortality, and there was no excess mortality between March and July, 2021, suggesting that the vaccine had no negative consequences, and protected the population from disease.

Less strict restrictions were in place during the fourth wave of COVID-19 which hit Israel hard with the Delta mutation of the COVID-19 virus, leading to high excess mortality between August and October, 2021 almost as high as the second wave for all population groups. Since most of population was vaccinated in the early months of 2021, the efficacy of their vaccinations seems to have been reduced after 5–6 months, and this together with the virulence of the Delta strain led to the high excess mortality. To overcome this, a booster shot was offered from August 2021, and this appears to have helped control the infections and reduce the excess mortality.

### Excess mortality by age

Significantly higher excess mortality was found only for ages above 55, particularly high for males aged 65–74 (11% higher), maybe because deaths are less frequent in general at this age and they seem to have been more susceptible to severe COVID-19. It is also possible that the excess mortality was lower or not much higher in the oldest population (85 and above or 75 and above) than younger groups (65–74, 75–84) because many of them were very careful to self–isolate as recommended by the Israeli government, sometimes at significant emotional cost! At ages under 45, there was no significant excess, and mortality was significantly lower for ages 0–24, which may be a result of the protective effects of lockdown, and lifestyle changes due to COVID-19, on mortality in youth, children and newborns. Evidence for this can be seen, for example, in the reduction of visits to the emergency room in 2020 for all diagnoses at ages 0–17, particularly in the early months of the pandemic [5]. Similarly there was a reduction in traffic accidents with injury and mortality in 2020 compared to 2019 at all ages, a 26% reduction in mortality at ages 0–14 [6].

### COVID-19 and non-COVID-19 mortality

Non-COVID-19 mortality was significantly lower during the whole period compared to total mortality in previous years in most age groups, and for the months of the third wave, particularly in January and February 2021, but already lower in November, 2020 and continuing to March 2021. Therefore, although total COVID-19 mortality was high in January and February 2021, as shown in Fig. 2 (the blue difference between total and non-COVID-19 mortality), the excess mortality was much lower than in the second wave, particularly among Jews and Others, only slightly higher than in the first wave.

The reason for this may be a result of the high numbers of COVID-19 deaths during the peaks of infection, with high mortality particularly at older ages. It is likely that many of the COVID-19 deaths in the second and third wave were among vulnerable people with serious preexisting medical conditions who may unfortunately have later died otherwise from their terminal illnesses. Indeed Baskaran et al. have shown in a British study that COVID-19 mortality was strongly associated with comorbidities as well as with increasing age [7]. The premature COVID-19 deaths of these people may have reduced the non-COVID-19 mortality over the period and, particularly in January and February, months with the highest mortality in Israel [8]. This “mortality displacement” effect was seen in June 2021 in an analysis of English mortality data [9].

The low non-COVID-19 mortality may also indicate that in Israel the population did not suffer significantly from lack of access to health services during lockdown. This is supported by the fact that the rate of doctor consultations (total of physical and virtual) in 2020 compared to 2019 did not decrease in Israel [10]. In this, Israel appears different from the USA where provisional data showed higher mortality rates from heart disease and diabetes in 2020 [11]. However, the final cause of death data in Israel for 2020 has not yet been published, to enable confirmation of this.

### Excess mortality in the Arab population

We found significantly higher excess mortality in the Arab population than Jews and Others, at most age groups and months of the pandemic, particularly in the third wave. When the low non-COVID-19 mortality in January–February, 2021 is taken into account, the COVID-19 deaths are likely to have been even higher.

Saban et al. have also reported on the excess morbidity in the Arab population in Israel in the first year of the pandemic and discussed its causes [12]. In other countries, too, it was noticed early on and confirmed by later research that ethnic minority groups, often of lower socioeconomic status, were most effected by the pandemic [7, 13, 14]. Among contributory factors suggested are their employment in occupations that do not allow social distancing or working from home and their increased use of public transport exposing them more to infection. Other reasons relevant to the Arab population are the frequent social interactions and large wedding celebrations, and the family centered Arab lifestyle, with multigenerational family homes exposing the elderly to infection acquired by the more exposed younger people. Further factors leading to increased infection may have been a somewhat slow uptake of the COVID-19 vaccination in the Arab sector and lack of adherence to protective guidelines, possibly due to distrust of the government.

Despite these factors, we note from Fig. 3 that the age-adjusted infection rate for the total Arab population had a lower peak in the first and third wave, and similar rates to the Jews and Others over the pandemic period. However, the older Arab population appear to have been less protected and infection rates at ages 65 and above were more than twice as high in the second and third waves. Risk of mortality from COVID-19 has been shown by Bhaskaran et al. [7] and by published mortality rates to be much higher at older ages. In addition, Bhaskaran et al. show increased risk with obesity, diabetes and smoking. The Arab population has a higher prevalence of obesity and diabetes than Jews and Others and higher smoking rates in males [15]. The combination of these higher infection rates at older ages and higher comorbid risk factors and disease can explain the much higher excess mortality in the total and older Arab population.

One factor unlikely to have increased Arab mortality is differential access to health services, mentioned as a possible factor in the USA [13], since COVID-19 testing and hospitalization was freely available to all the population through comprehensive national health insurance and a countrywide network of hospitals. However, there were some small private health initiatives in the Jewish and particularly Orthodox populations to enable home care instead of hospitalization by provision of life-saving equipment and care [16]. This may have helped lower Jewish mortality, particularly at the peaks of infection when hospital COVID-19 wards were overcrowded and understaffed.

Significantly higher non-COVID-19 mortality was found for Arabs in August and September 2021, and also in May and September 2020, and for Jews and Others only in August 2021. This could be a result of under-reporting of COVID-19 infections, leading to deaths incorrectly being recorded as non-COVID-19, or due to a true increase in non-COVID-19 deaths in these months, maybe following a lowering of their level of routine healthcare, possibly as a side consequence of the pandemic.

### Mortality in a vaccinated cohort

We saw no evidence of a deleterious effect of the vaccine as shown by mortality in the cohort of persons vaccinated at least once, over a seven-month period. On the contrary, we found lower mortality rates. The lower rates may be due to the vaccinated cohort being a healthier population, since vulnerable people with serious preexisting medical conditions may have died from COVID-19 before the vaccination became available, or because they did not vaccinate. It could also be a result of generally falling death rates, or behavioral protective changes in persons who vaccinated and their tendency to choose healthier lifestyle options. There were some deaths attributed to COVID-19, as a result of breakthrough infections some of which may have occurred after one vaccination only, but a relatively small number. Low non-COVID-19 mortality risk was also found in a study of vaccinated in the USA, but the comparison was of mortality of vaccinated persons compared to non-vaccinated [17].

The Arab population may have been under-represented in the vaccinated cohort since their uptake of the vaccine was later, which may have influenced the results.

### Health policy implications

The high excess mortality we showed during the COVID-19 waves highlights the importance of preventing infection as much as possible, such as by mandating mask wearing, encouraging social distancing and keeping population immunity up-to-date with booster vaccinations.

We found that in Israel the excess mortality was similar to the deaths attributed to COVID-19 mortality, unlike in many other countries [18]. This validates the widespread and free testing for COVID-19 allowing identification of those who were infected, and the speedily created infrastructure for complete COVID-19 databases which enabled a follow-up of the consequences of the infection, including mortality.

## Limitation

This study is based on total mortality data in Israel available at time of writing, which is assumed to be complete through October 2021. Minor adjustments could occur to the numbers of deaths, but are unlikely to change the results in a major way.

Due to errors in a small number of identification numbers in the vaccination file, the matching of the vaccination and death file may have been erroneous for a small minority of records in the file.

## Conclusion

Israel has seen significant excess mortality in four waves of COVID-19, particularly in the Arab sector. Lockdowns and vaccinations appear to have helped in controlling the excess mortality, and no excess mortality was seen in the first 5 months after the vaccination campaign. It is important to continue public health measures such as mandating mask wearing and population vaccinations to control infection and reduce excess mortality.

## Supplementary Information

**Additional file 1: Table S1.** Ratio of mortality rates for the period March 2020–October 2021, for all deaths and non-COVID-19 deaths, compared to average of 2017–2019, by month and population group with 95% CI. **Table S2.** Ratio of mortality rates for the period March 2020–October 2021, for all deaths and non-COVID-19 deaths, compared to average of 2017–2019, by age groups, sex, and population group with 95% CI. **Table S3.** Ratio of mortality rates for the period April–October 2021 of a cohort of persons vaccinated before 31 March 2021, aged 15 and above, compared to the average mortality rates in April–October 2017–2019, by age and sex with 95% CI.

## Data Availability

Data on mortality and causes of death in Israel by year are available from the WHO mortality database. Data on COVID-19 tests, cases and deaths are available from the Israeli government COVID-19 repository [19].

## Abbreviation

COVID-19: Corona virus disease 2019
